# Molecular Networking-Based Metabolome, In Vitro Antidiabetic and Anti-Inflammatory Effects of *Breonadia salicina* (Vahl) Hepper & J.R.I. Wood

**DOI:** 10.3390/metabo14060291

**Published:** 2024-05-21

**Authors:** Dorcas Tlhapi, Isaiah Ramaite, Chinedu Anokwuru, Teunis van Ree

**Affiliations:** 1 Department of Chemistry, Faculty of Science, Engineering and Agriculture, University of Venda, Private Bag X5050, Thohoyandou 0950, South Africa; isaiah.ramaite@univen.ac.za (I.R.); teuns.vanree@univen.ac.za (T.v.R.); 2 Department of Basic Sciences, School of Science and Technology, Babcock University, Ilishan Remo 121103, Nigeria; anokwuruc@babcock.edu.ng

**Keywords:** *Breonadia salicina*, UPLC-QTOF-MS, molecular networking, phytochemical compounds, antidiabetic activity, anti-inflammatory activity, antiproliferation activity, genotoxicity, cytotoxicity

## Abstract

*Breonadia salicina* (Vahl) Hepper & J.R.I. Wood is widely distributed throughout Africa. It is used ethnobotanically to treat various diseases. However, the metabolic profile of the *Breonadia* species is not well characterized and the metabolites that are responsible for the bioactivity of this plant remain unknown. Therefore, there is a need to determine the phytochemical and bioactivity profile to identify metabolites that contribute to the antidiabetic, anti-inflammatory and antiproliferation activity, including the genotoxicity and cytotoxic effects, of *Breonadia salicina*. The study is aimed at exploring the metabolomic profile antidiabetic, anti-inflammatory and antiproliferation activity, as well as the genotoxicity and cytotoxicity effects, of constituents of *B. salicina*. The compounds in the *B. salicina* extract were analyzed by ultra-performance liquid chromatography with quadrupole time-of-flight mass spectrometry (UPLC-QTOF-MS), and the resultant data were further analyzed using a molecular networking approach. The crude stem bark and root extracts showed the highest antidiabetic activity against α-amylase at the lowest test concentration of 62.5 µg/mL, with 74.53 ± 0.74% and 79.1 ± 1.5% inhibition, respectively. However, the crude stem bark and root extracts showed the highest antidiabetic activity against α-glucosidase at the lowest test concentration of 31.3 µg/mL, with 98.20 ± 0.15% and 97.98 ± 0.22% inhibition, respectively. The crude methanol leaf extract showed a decrease in the nitrite concentration at the highest concentration of 200 µg/mL, with cell viability of 90.34 ± 2.21%, thus showing anti-inflammatory activity. No samples showed significant cytotoxic effects at a concentration of 10 µg/mL against HeLa cells. Furthermore, a molecular network of *Breonadia* species using UPLC-QTOF-MS with negative mode electrospray ionization showed the presence of organic oxygen compounds, lipids, benzenoids, phenylpropanoids and polyketides. These compound classes were differentially distributed in the three different plant parts, indicating the chemical differences between the stem bark, root and leaf extracts of *B. salicina.* Therefore, the identified compounds may contribute to the antidiabetic and anti-inflammatory activity of *Breonadia salicina*. The stem bark, root and leaf extracts of *B. salicina* yielded thirteen compounds identified for the first time in this plant, offering a promising avenue for the discovery of new lead drugs for the treatment of diabetes and inflammation. The use of molecular networking produced a detailed phytochemical overview of this *Breonadia* species. The results reported in this study show the importance of searching for bioactive compounds from *Breonadia salicina* and provide new insights into the phytochemical characterization and bioactivity of different plant parts of *Breonadia salicina*.

## 1. Introduction

*Breonadia salicina* (Vahl) Hepper and J.R.I. Wood is a monotypic genus of flowering plants in the family Rubiaceae [1]. *Breonadia salicina* was described by Colin Ernest Ridsdale in 1975 [2]. This species is a protected tree in South Africa and is often used in traditional medicine [1]. The aerial and underground parts of the plant are often used in treating various diseases [3,4,5,6]. Ethnomedicinal information reveals that this plant is commonly used by traditional healers in South Africa and other African countries for the treatment of diabetes and inflamed wounds due to its phytochemical constituents [7]. Although researchers have been investigating the chemical composition and bioactivity of *Breonadia salicina*, the chemical profile of this species is not well explained; the bioactive compounds are thus unknown. Studies on the phytochemistry of *Breonadia salicina* have revealed very few isolated compounds, such as chlorogenic acid (5-*O*-caffeoylquinic acid), a flavonoid (kaempferol 3-*O*-(2″-*O*-galloyl)-glucuronide), a monosaccharide (D-galactopyranose), an oleanane triterpenoid saponin (bodinioside Q), a disaccharide (sucrose), saturated fatty acids (hexadecane and palmitic acid), 7-(β-D-apiofuranosyl(1–6)-β-D-glucopyranosyl) umbelliferone and 6-hydroxy-7-methoxycoumarin, using a reductionist approach [3,6,8,9]. According to a literature survey, the antidiabetic and anti-inflammatory activity of *B. salicina* has never been evaluated. Therefore, there is a need to determine which phytochemicals contribute to the antidiabetic and anti-inflammatory activity of *B. salicina*. The aim of this study was to investigate the in vitro antidiabetic and anti-inflammatory potential of *Breonadia salicina*. Furthermore, this study provides information on which part of the plant can be a potential source of safe and effective compounds for the treatment of diabetes and inflammation by using the α-amylase inhibition and α-glucosidase inhibition, Griess, cell toxicity, MTT (3-(4,5-dimethylthiazol-2-yl)-2,5-diphenyltetrazolium bromide), Hoechst 33342/propidium iodide (PI) dual staining and NucRed nuclei dye assay methods. This study also reveals the differences in the biological activity of extracts from various parts of a plant. The biological activity of these extracts could be due to the presence of a variety of low-molar-mass components that can contribute to their activity to varying degrees, such as secondary metabolites (phenols, carbohydrates, terpenoids and flavonoids), essential oils (and their components, like eugenol, benzaldehyde, thymol and α-tocopherol) and polyphenolic compounds. According to literature reports, the presence of these compounds can be influenced by the extraction method, and the varying concentrations of these compounds can lead to observed differences in biological activity when comparing extracts from different parts of the same plant [10].

Metabolomics is a term denoting the wide-ranging quantitative and qualitative analysis of a group of metabolites (targeted approach) or all metabolites (untargeted approach), classifying and quantifying the biomarkers in a complex matrix [11,12]. While the targeted approach focuses on analyzing a group of known metabolites, the untargeted approach involves the identification of numerous unexpected metabolites [13]. The identification and quantitation of secondary plant metabolites is supported mostly by liquid chromatography and gas chromatography coupled to mass spectrometry, NMR spectroscopy and FTIR spectroscopy [14,15,16]. However, liquid chromatography–mass spectrometry (LC-MS) is the most frequently used technology in metabolomics for the analysis of more polar constituents [17]. Furthermore, GC-MS is also a commonly used technique in metabolomics, used to detect trace quantities of targeted or untargeted metabolites that are non-polar, volatile or highly volatile [18,19]. Moreover, NMR spectroscopy is normally used as an analytical tool for plant metabolomics, to identify and characterize the structures of compounds [20]. Proton (^1^H) NMR spectroscopy is the most commonly applied technique in metabolomics [21]. The complete characterization of a compound is achieved with 2D-NMR [20]. Molecular networking is a tool used to identify and classify the similarity between metabolites [22]. This approach allows for the visual representation of the structural relationships among the natural constituents in crude extracts and further compares the tandem MS spectra to the publicly accessible spectral library for the rapid characterization and identification of known constituents [23]. A few investigations of the compounds in *B. salicina* have employed ^1^H-NMR and UPLC-QTOF-MS analysis—for example, for the characterization and identification of the metabolites contributing to the antioxidant [9], antimalarial and antitrypanosomal activity [24]. Liquid chromatography–mass spectrometry and UPLC-QTOF-MS techniques can only tentatively suggest the identities of metabolites, while the analysis of the NMR data of the isolated pure compounds provides the exact chemical structures of bioactive natural products. As mentioned above, a few studies have shown that *B. salicina* is used to treat diabetes; however, the active compounds in this plant have rarely been isolated and characterized using a reductionist approach. Furthermore, there are no reports on the chemical profiling of the bioactive extracts from *Breonadia* species toward α-glucosidase inhibition using a metabolomic approach. Applying these techniques, the stem bark, root and leaf extracts of *Breonadia salicina* were analyzed to identify possible antidiabetic and anti-inflammatory compounds.

## 2. Materials and Methods

### 2.1. General Experimental Procedure

All reagents and chemicals used for the biological assays were purchased from Sigma-Aldrich (St. Louis, MO, USA) and freshly prepared for these assays. All chemicals used for the UPLC-QTOF-MS analysis were of analytical grade, purchased from Sigma-Aldrich (Darmstadt, Germany). A liquid chromatography–quadrupole time-of-flight tandem mass spectrometer (LC-MS-9030 q-TOF, Shimadzu Corporation, Kyoto, Japan) was fitted with a Shim-pack Velox C_18_ column. The solvents used for the chromatographic runs were formic acid and methanol, which were purchased from Sigma-Aldrich (Darmstadt, Germany).

### 2.2. Sampling and Extraction

The stem bark, root and leaf samples of *Breonadia salicina* were gathered in October 2019 at Fondwe, a village in Limpopo Province, at a 22°55′31.9″ south latitude and 30°15′45.0″ east longitude. The samples were identified at the Department of Biological Science, University of Venda, by Prof. Peter Tshisikhawe. Voucher sample BD 02 was placed in the department’s herbarium. After four weeks of air drying, the samples were ground with a hammer mill (NETZSCH, Selb, Germany). The ground stem bark (about 1.74 kg) and 1.01 kg ground root samples were each soaked in 2 L MeOH for 48 h at ambient temperature. After filtration and evaporation at 45 °C in a rotary evaporator (BÜCHI Labortechnik AG, Flawil, Switzerland), 95.57 g crude ground stem bark extract and 68.82 g crude root extract were obtained. Furthermore, about 1.12 kg ground *B. salicina* leaves were soaked successively at room temperature in 2 L dichloromethane (DCM) followed by MeOH, each for 48 h. After filtration and evaporation at 45 °C, 20.52 g crude DCM extract and 66.43 g crude MeOH extract were obtained [9].

### 2.3. UPLC-QTOF-MS Analysis

A liquid chromatograph–quadrupole time-of-flight tandem mass spectrometer (LC-MS-9030 q-TOF, Shimadzu Corporation, Kyoto, Japan) was fitted with a Shim-pack Velox C_18_ column (100 mm × 2.1 mm with particle size of 2.7 μ). The column oven temperature was maintained at 50 °C. The injection volume was 5 µL and the samples were analytically separated over a 30 min binary gradient. The flow rate was kept constant at 0.3 mL/min in the current study using a binary solvent mixture of water with 0.1% formic acid (Eluent A) and methanol with 0.1% formic acid (Eluent B). The gradient technique was gradually increased from 3 to 30 min to facilitate the separation of the compounds within the samples. Briefly, Eluent B was kept at 5% from 0 to 3 min, gradually increased from 5 to 40% between 3 and 8 min and finally increased to 40–95% between 8 and 23 min. Eluent B was then kept isocratic at 95% between 23 and 25 min. The gradient was returned to the original conditions of 5% at 25–27 min, and re-equilibration at 5% occurred at 27–30 min. The liquid chromatographic eluents were then subjected to a quadrupole time-of-flight high-definition mass spectrometer for analysis in negative ESI mode. The q-TOF-MS conditions were as follows: 400 °C heat block temperature, 250 °C desolvation line (DL) temperature, 42 °C flight tube temperature and 3 L/min nebulization and a dry gas flow. In the data-dependent (DDA) mode, MS1 and MS2 were acquired simultaneously for all ions, with an intensity threshold above 5000 at an *m/z* range of 100–2000. The MS2 experiments were conducted utilizing argon gas as the collision gas and a collision energy of 30 eV with a spread of 5. Sodium iodide (NaI) was used as a calibration solution to ensure high mass precision.

### 2.4. Global Natural Product Social Molecular Networking (GNPS) and Metabolite Annotation

Molecular networks were generated on the Global Natural Product Social Molecular Networking (GNPS) platform (http://gnps.ucsd.edu (accessed on 16 January 2024)) to explore the chemistry of various plant parts of *Breonadia salicina*. The raw data were converted to an open-source format (.mzML) and the spectral data were uploaded to WinSCP, which can be used to store and transfer data online. Once the spectral data were uploaded, the molecular networking connected the mass spectra of molecules based on the similarity of their fragmentation ions/patterns. The output of the molecular network was visualized using Cytoscape version 3.9.1 [25]. For compound annotations, the empirical formulas generated from the accurate mass obtained from the MS/MS data of the compounds were used to annotate the matched and some unmatched nodes, and these were compared to other common natural product dereplication databases, such as KNApSAck (www.knapsackfamily.com (accessed on 13 February 2024)), the Dictionary of Natural Products (http://dnp.chemnetbase.com/faces/chemical/ChemicalSearch.xhtml (accessed on 11 March 2024)) and ChemSpider (www.chemspider.com (accessed on 8 April 2024).

### 2.5. Antidiabetic Activity

#### 2.5.1. α-Amylase Inhibition Assay

The crude extracts were reconstituted in dimethyl sulfoxide (DMSO; Sigma-Aldrich, St. Louis, MO, USA) to a concentration of 100 μg/mL. The samples were diluted in assay buffer (Dulbecco’s Phosphate-Buffered Saline (PBS)) to concentrations of 500, 250, 125 and 62.5 μg/mL. Fifteen µL of each extract was added to 96-well plates in quadruplicate, followed by 5 μL enzyme (Sigma-Aldrich, St. Louis, MO, USA). The 96-well plates were incubated at 37 °C for 10 min. After incubation, 20 μL starch solution was added to the 96-well plates. The plates were further incubated at 37 °C for 30 min. Ten microliters of stop solution and 75 μL iodine reagent (Sigma-Aldrich, St. Louis, MO, USA) were added to the 96-well plates [26]. The absorbance was measured at 580 nm using a BioTek^®^ PowerWave XS spectrophotometer (Winooski, VT, USA).

#### 2.5.2. α-Glucosidase Inhibition Assay

The crude extracts were reconstituted in dimethyl sulfoxide (DMSO; Sigma-Aldrich, St. Louis, MO, USA) to a final concentration of 100 µg/mL. The samples were diluted in assay buffer (67 mM potassium monobasic anhydrous phosphate at pH 6.8, with 3 mM reduced glutathione) to concentrations of 500, 250, 125, 62.5 and 31.3 μg/mL. Five µL of each extract was added to 96-well plates in quadruplicate, followed by 20 μL enzyme (Sigma-Aldrich, St. Louis, MO, USA). Sixty microliters of buffer was added to the solution in the 96-well plates and they were incubated at 37 °C for 5 min. After incubation, 10 μL p-NP-Gluc was added. Thereafter, the 96-well plates were incubated at 37 °C for 20 min and 25 μL Na_2_CO_3_ (Sigma-Aldrich, St. Louis, MO, USA) was added [27,28]. The absorbance was measured at 405 nm using a BioTek^®^ PowerWave XS spectrophotometer (Winooski, VT, USA).

### 2.6. Anti-Inflammatory Activity

The crude extracts were solubilized using dimethyl sulfoxide (DMSO; Sigma-Aldrich, St. Louis, MO, USA) to produce a stock solution of 100 mg/mL. Aminoguanidine (100 μM; Sigma-Aldrich, St. Louis, MO, USA) was used as a positive control to indicate the anti-inflammatory activity. RAW 264.7 cells (Cellonex, South Africa) were seeded in RPMI1640 culture medium supplemented with 10% FBS (RPMI complete medium; GE Healthcare Life Sciences, Logan, UT, USA) into 96-well plates at a density of 1 × 10^5^ cells per well and allowed to attach overnight. The following day, the spent culture medium was removed and 100 µL aliquots of each extract (diluted in RPMI complete medium) were added to give final concentrations of 50, 100 and 200 μg/mL. To assess the anti-inflammatory activity, 100 μL of LPS (final concentration 500 µg/mL; Sigma-Aldrich, St. Louis, MO, USA) containing the medium was added to the corresponding wells. Aminoguanidine (AG; Sigma-Aldrich, St. Louise, MO, USA) was used as the positive control at 100 μM. Cells were incubated for a further 24 h. To quantify NO production, 50 μL of the spent culture medium was transferred to a new 96-well plate and 50 μL Griess reagent (Sigma-Aldrich, St. Louise, MO, USA) was added [29]. The absorbance was measured at 540 nm. A standard curve using sodium nitrite dissolved in the culture medium was used to determine the concentration of NO in each sample.

### 2.7. Antiproliferation Activity

To evaluate the cytotoxicity, the crude extracts were incubated at fixed concentrations of 50 μg/mL and 10 μg/mL, respectively, in 96-well plates seeded 24 h earlier against HeLa cells (Cellonex; 2 × 10^4^ cells per well). Incubation was carried out for 48 h in a 37 °C 5% CO_2_ humidified incubator. Dulbecco’s modified Eagle’s medium (DMEM; Thermo Fisher Scientific) containing the culture medium was added with ten percent (%) of fetal bovine serum (Biowest) and penicillin/streptomycin/amphotericin (Lonza). Furthermore, resazurin (Sigma-Aldrich) was added to a final concentration of 0.05 mM, followed by incubation for 2 h. After incubation, the fluorescence (Exc560/Em590) was determined using a Spectramax M3 plate reader (Molecular Devices) (San Jose, CA, USA) [30]. The fluorescence values were converted into the percentage (%) of cell viability relative to the untreated control, after subtracting background readings obtained from wells without cells.

### 2.8. Genotoxicity

The crude extracts were reconstituted in DMSO to give a final concentration of 100 mg/mL. The extracts were stored overnight at 4 °C and the cells were seeded in 96-well plates at 3000 cells/well (100 μL aliquots). Cells were treated with 15.125, 31.25, 62.5, 125 and 250 μg/mL extract for 48 h at 37 °C under an atmosphere of 5% CO_2_. Griseofulvin was used as the positive control at a concentration range of 0–50 μM. After incubation, cells were fixed using 4% formaldehyde for 15 min. NucRed working solution was prepared by adding 2 drops of NucRed per mL complete medium. The fixative was aspirated prior to the addition of 100 μL NucRed working solution. Cells were stained for 15–30 min and thereafter images were acquired using the Cy5 filter on the ImageXpress Micro XLS Widefield Microscope (Molecular Devices). The quantification of live and dead cells for the screening assay was performed using the ImageXpress Micro XLS Widefield Microscope (Molecular Devices) and the acquired images analyzed using the MetaXpress software and Multi-Wavelength Cell Scoring Application Module. The acquired data were transferred to an EXCEL spreadsheet and further processed [31,32,33].

### 2.9. Cytotoxicity

#### 2.9.1. MTT Assay

The cell viability of the crude extracts was assessed using MTT (3-(4,5-dimethylthiazol-2-yl)-2,5-diphenyltetrazolium bromide) against RAW 264.7 macrophages in order to accurately establish the potential anti-inflammatory activity and to confirm the absence of toxicity as a contributory factor. The remaining medium and treatments in each 96-well plate were removed and replaced with medium containing 0.5 mg/mL MTT (3-(4,5-dimethylthiazol-2-yl)-2,5-diphenyltetrazolium bromide). Thereafter, the 96-well plates were incubated for 30 min at 37 °C. After incubation, the 3-(4,5-dimethylthiazol-2-yl)-2,5-diphenyltetrazolium bromide was removed and 100 μL DMSO was added to each well to solubilize the formazan crystals [34]. The absorbance was read at 540 nm using a BioTek^®^ PowerWave XS spectrophotometer.

#### 2.9.2. Hoechst 33342/Propidium Iodide (PI) Dual Staining Method

The cytotoxicity of the stem bark, root, methanol leaf and dichloromethane leaf extracts was assessed using the Hoechst 33342/propidium iodide (PI) dual staining method. The extracts were reconstituted in DMSO to give a final concentration of 100 mg/mL. Thereafter, 100 μL cells (4000 Vero cells/well density) were seeded into 96-well microtiter plates. The microtiter plates were incubated at 37 °C, in 5% CO_2_ and at 100% relative humidity for 48 h. Furthermore, a 100 μL aliquot of each dilution was added to 100 μL of attached cells in the 96-well plate at concentrations of 15.625, 31.25, 62.5, 125 and 250 µg/mL. The plates were incubated for 30 min and 100 μL of the Hoechst 33342 nuclear dye staining solution was added to each well. Finally, 10 μL propidium iodide (PI) solution [35] was added to each well. The quantification of live and dead cells was performed using the ImageXpress Micro XLS Widefield Microscope (Molecular Devices) using a 10× Plan Fluor objective and DAPI and Texas Red filter cubes. Melphalan (100 mM stock) was used as a positive control at 30 μM. The cytotoxicity of the extracts was assessed using the Hoechst 33342/propidium iodide (PI) dual staining method.

### 2.10. Statistical Analysis

For the α-amylase inhibition assay, the data for the tested crude extracts were shown as the percentage (%) of α-amylase inhibition ± standard deviation (SD). Similarly, for the α-glucosidase inhibition assay, the data for the tested crude extracts were shown as the percentage (%) of α-glucosidase inhibition ± standard deviation (SD). For the anti-inflammatory activity, the data for the tested crude extracts were shown as nitric oxide production ± standard deviation (SD). Acarbose was used as a positive control for the α-amylase inhibition assay, epigallocatechin gallate (ECGC) was used as a positive control for the α-glucosidase inhibition assay and aminoguanidine was used as a positive control to indicate anti-inflammatory activity. For the antiproliferation activity, the data for the single concentration assay were shown as the percentage (%) of parasite viability ± standard deviation (SD). Emetine (which induces cell apoptosis) was used as a positive control drug standard for antiproliferation activity. For genotoxicity, the data for the cytotoxicity were shown as the Vero cell number ± standard deviation at varying concentrations, while the data for the micro-nucleated cells were shown as the percentage of micro-nucleated Vero cells ± standard deviation at varying concentrations. Griseofulvin was used as a positive control for the genotoxicity screening. Furthermore, for the MTT assay, the data for the cytotoxicity were shown as the percentage of cell viability of LPS-activated macrophages ± standard deviation at varying concentrations. Moreover, for the cytotoxicity assessed using the Hoechst 33342/propidium iodide (PI) dual staining method, the data were shown as the average number of live cells ± standard deviation at varying concentrations.

## 3. Results and Discussion

### 3.1. Molecular Networking of Breonadia Metabolites

**Figure 1:**
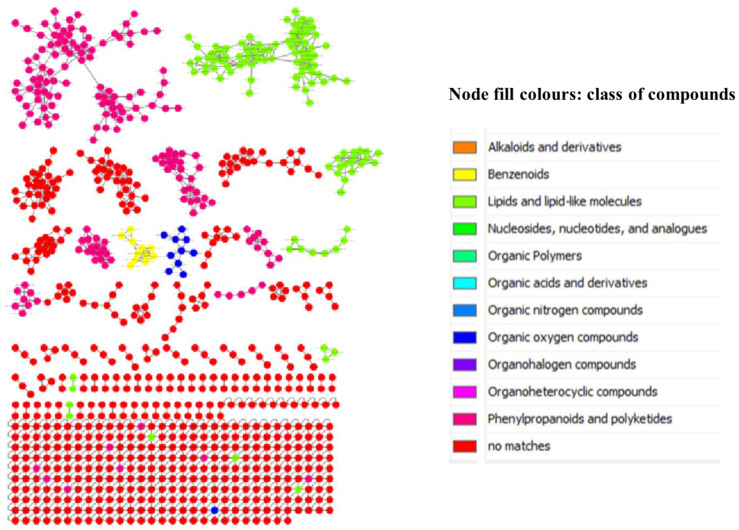
An enhanced molecular network of crude extracts from *Breonadia* species in which nodes are colored based on their chemical superclass, as analyzed by UPLC-QTOF-MS using electrospray ionization in negative mode, with phenylpropanoids, polyketides, lipids, organic oxygen compounds and benzenoids as the major metabolite class identities.

**Figure 2:**
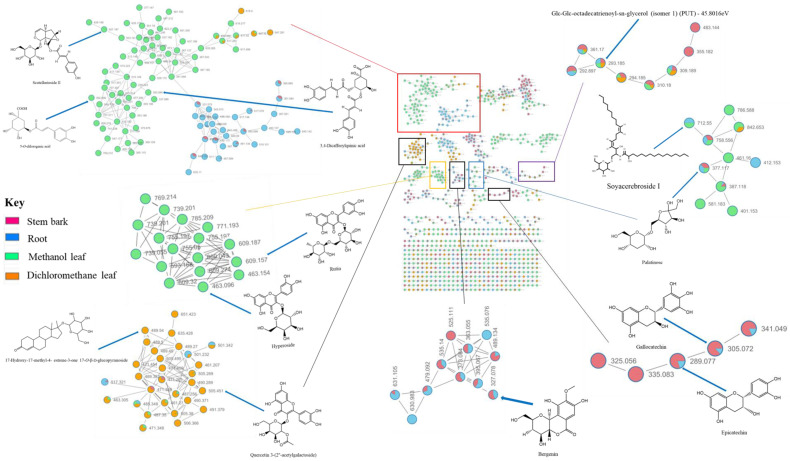
A magnified molecular network of *Breonadia salicina* crude extracts. Nodes from the selected molecular class are labeled with the precursor mass and displayed as pie charts that represent the distribution of the ion intensities of the stem bark (red), root (blue), dichloromethane leaf (orange) and methanol leaf (green) crude extracts.

To explore the chemistries of *Breonadia* species, the stem bark, root and leaf extracts of *Breonadia salicina* were analyzed using UPLC-QTOF-MS operating in negative mode (ESI -), and the spectral data were acquired using the data-dependent acquisition (DDA) protocol, which generates MS^2^ fragmentation data for every precursor ion over a prearranged threshold. The UPLC-QTOF-MS chromatograms of the *B. salicina* crude extracts are provided in Figure S1 (Supplementary Materials). Furthermore, the chemical variety of the stem bark, root and leaf extracts of *B. salicina* was studied through a publicly accessible database via the classical molecular networking workflow on the GNPS platform, allowing a comprehensive overview of the chemical information deduced from the MS/MS data. The generated molecular networks were visualized in Cytoscape version 3.9.1 (as shown in Figure 1, [25]). An enhanced classical molecular network generated with tools such as in silico annotation tools (network annotation propagation (NAP) and DEREPLICATOR^+^) and an unsupervised substructure identification tool (MS2LDA) were also studied to complete the classical molecular networking output and integration using MolNetEnhancer within GNPS. The computed classical molecular network (MN) consisted of 894 nodes connected through 1460 edges with 577 cluster nodes in the network (as seen in Figure 1). The enhanced network allowed the annotation of numerous known and unknown metabolites belonging to different chemical classes, such as phenylpropanoids, polyketides, lipids, organic oxygen-containing compounds and benzenoids. According to some literature studies, the antidiabetic activity of various plant species is due to the presence of natural inhibitors such as benzenoids, phenylpropanoids, polyketides, lipids (fatty acids) and organic oxygen-containing compounds [36]. Furthermore, studies have shown that lipids (fatty acids), phenylpropanoids and polyketides are responsible for the anti-inflammatory action of various plant species [37]. Therefore, the phytochemicals discovered from *Breonadia* species may be useful for the treatment of diabetes and inflammation. To further evaluate the influence of different plant parts on the molecular network topology, the major classes of compounds (phenylpropanoids, polyketides, lipids, benzenoids and organooxygen compounds) were magnified (Figure 2). This was to study the chemical variety and compare the metabolome compositions of the stem bark, root and leaf extracts of *Breonadia salicina*. In the magnified networks, the computed classical molecular network (MN) consisted of 894 nodes connected through 1460 edges. The methanol leaf extracts had the highest number of nodes (346), followed by the stem bark (200 nodes), root (198 nodes) and dichloromethane leaf extracts (150 nodes). The methanol leaf extracts solely contained many metabolites compared to the stem bark, root and dichloromethane leaf extracts. Furthermore, a combination of molecular network and manual confirmatory scrutiny led us to identify unique compounds within the stem bark, root and leaf extracts of *B. salicina*. A metabolite at *m*/*z* 293.19, annotated as Glc-Glc-octadecatrienoyl-sn-glycerol (isomer 1), was present in all plant parts (stem bark, root, dichloromethane leaf and methanol leaf), whereas the metabolites at *m*/*z* 505.26 and *m*/*z* 489.54, annotated as quercetin 3-(2″-acetylgalactoside) and 17-hydroxy-17-methyl-4-estren-3-one 17-*O*-β-D-glucopyranoside, were only found in the dichloromethane leaf extracts. However, the metabolites at *m*/*z* 305.07, *m*/*z* 289.07 and *m*/*z* 327.07, annotated as gallocatechin, epicatechin and bergenin, respectively, were only present in the stem bark and root extracts. Moreover, a metabolite at *m*/*z* 377.11, annotated as palatinose, was found in the stem bark, root and methanol leaf extracts, whereas a metabolite at *m*/*z* 712.55, annotated as soyacerebroside I, was only present in the root and methanol leaf extracts. The methanol leaf extracts contained unique metabolites at *m*/*z* 463.09, *m*/*z* 507.19, *m*/*z* 354.09, *m*/*z* 609.15 and *m*/*z* 353.09, annotated as hyperoside, scutellarioside II, 5-*O*-caffeoylquinic acid, rutin and 3,4-dicaffeoylquinic acid. The molecular network showed that the chemistry of the leaf extracts differed from that of the root and stem bark extracts. In addition, the chemistries of the methanol and dichloromethane leaf extracts were different.

### 3.2. Antidiabetic Activity

**Figure 3:**
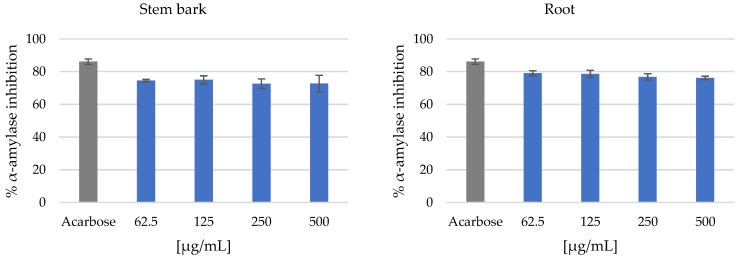

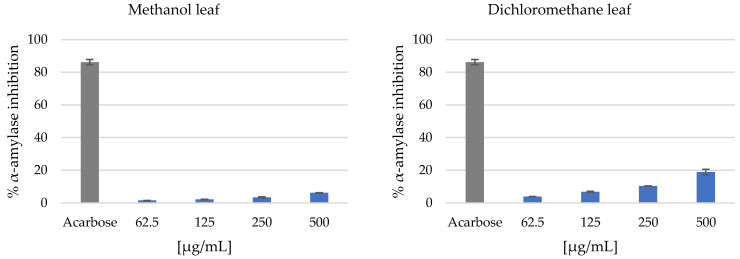
α-Amylase inhibition assay: stem bark crude extract, root crude extract, methanol leaf crude extract and dichloromethane leaf crude extract. Data (n = 4) expressed as percentage α-amylase inhibition ± standard deviation at varying concentrations.

**Figure 4:**
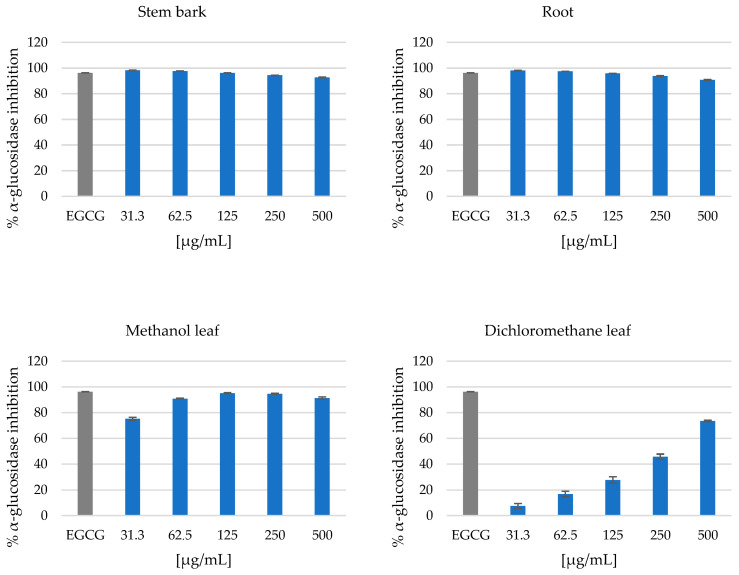
α-Glucosidase inhibition assay: stem bark crude extract, root crude extract, methanol leaf crude extract and dichloromethane leaf crude extract. Data (n = 4) expressed as percentage α-glucosidase inhibition ± standard deviation at varying concentrations.

For the α-amylase inhibition assay, the stem bark and root extracts showed very strong activity at the lowest test concentration of 62.5 µg/mL, with inhibition of 74.53 ± 0.74% and 79.1 ± 1.5%, respectively, as shown in Figure 3. Furthermore, the methanol and dichloromethane leaf extracts did not exhibit any noteworthy activity at the lowest test concentration of 62.5 µg/mL, with inhibition of 1.532 ± 0.115% and 3.864 ± 0.115%, respectively. However, the crude dichloromethane leaf extract showed slight inhibition of <20% observed at 500 µg/mL, as shown in Figure 3. For the α-glucosidase inhibition assay, the stem bark and root extracts showed the complete inhibition of α-glucosidase at the lowest test concentration of 31.3 µg/mL at 98.20 ± 0.15% and 97.98 ± 0.22%, respectively, as shown in Figure 4. Furthermore, the crude methanol leaf extract exhibited strong inhibitory activity at a concentration of 125 µg/mL at 95.16 ± 0.41%. However, the crude dichloromethane leaf extract displayed low inhibitory activity at a concentration of 125 µg/mL at 27.75 ± 2.39%, as presented in Figure 4. A concentration-dependent increase in inhibitory activity was observed for the dichloromethane leaf extract, with the highest inhibitory activity observed at a test concentration of 500 µg/mL at 73.46 ± 0.52%, as shown in Figure 4. However, the stem bark, root and methanol leaf extracts showed good α-glucosidase inhibition activity at a concentration of 500 µg/mL at 92.66 ± 0.23%, 90.66 ± 0.33% and 91.33 ± 0.89%, respectively, as shown in Figure 4.

The presence of the identified lipids (glc-glc-octadecatrienoyl-sn-glycerol (isomer 1) and scutellarioside II), benzenoids (bergenin), organic oxygen compounds (palatinose and soyacerebroside I), phenylpropanoids and polyketides (hyperoside, gallocatechin, epicatechin, 5-*O*-caffeoylquinic acid, rutin and 3,4-dicaffeoylquinic acid) might have contributed to the potent antidiabetic activity in the stem bark, root and methanol leaf extracts, as shown in Figure 1 and Figure 2. Nkobole et al. (2011) reported that epicatechin and gallocatechin, tentatively identified from the stem bark and root crude extracts (as shown in Figure 2), exhibited high antidiabetic activity against α-glucosidase and α-amylase, with inhibition concentrations of 255.8 µM and 304.9 µM, respectively, from a *Terminalia sericea* stem bark extract [38]. However, San et al. (2020) reported that bergenin, isolated from the root extracts of *Cissus javana* DC. (Vitaceae), had antidiabetic activity, as measured by α-glucosidase inhibition at 100 μg/mL (0.3046 mM) [39]. Astiti et al. (2021) showed that rutin isolated from the leaf crude extracts of *Coccinia grandis* had potent antidiabetic activity as shown by α-glucosidase inhibition, with an IC_50_ value of 243.4 µM [40]. Furthermore, there are no reports or studies on the in vitro and in vivo antidiabetic activity of glc-glc-octadecatrienoyl-sn-glycerol (isomer 1), palatinose, soyacerebroside I, scutellarioside II, 5-O-caffeoylquinic acid, hyperoside and 3,4-dicaffeoylquinic acid, which were also identified in the stem bark, root and methanol leaf extracts (as presented in Figure 1 and Figure 2). Furthermore, our study is the first to detect the important antidiabetic activity of *Breonadia salicina*.

### 3.3. Anti-Inflammatory Activity

**Figure 5:**
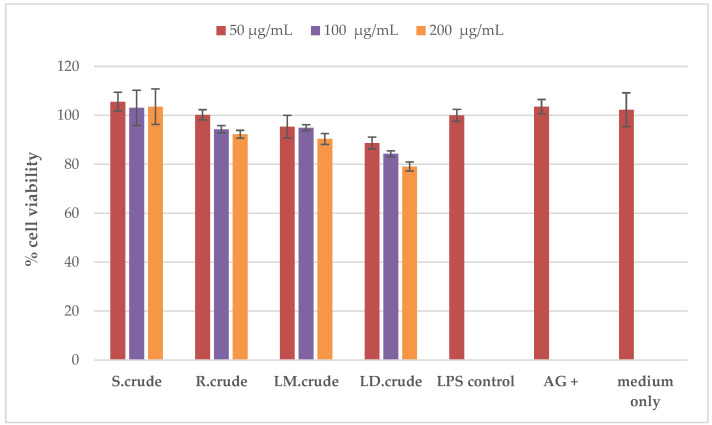
Anti-inflammatory activity against RAW 264.7 macrophages: S.crude—stem bark crude extract; R.crude—root crude extract; LM.crude—methanol leaf crude extract; and LD.crude—dichloromethane leaf crude extract. Expressed as nitric oxide production ± standard deviation at varying concentrations.

The anti-inflammatory activity of the stem bark, root, methanol leaf and dichloromethane leaf extracts was assessed using RAW 264.7 macrophages and the Griess assay. The crude methanol leaf extract showed a decrease in the nitrite concentration at the highest concentration of 200 µg/mL, with cell viability of 90.34 ± 2.21%, as shown in Figure 5. Therefore, the presence of the identified metabolites, such as (glc-glc-octadecatrienoyl-sn-glycerol (isomer 1), quercetin 3-(2″-acetylgalactoside) and 17-hydroxy-17-methyl-4-estren-3-one 17-*O*-β-D-glucopyranoside, might have contributed to the strong anti-inflammatory activity of the crude dichloromethane leaf extract, as shown in Figure 2 and Figure 5. According to a literature survey, the anti-inflammatory activity of these identified metabolites from *Breonadia salicina* has never been evaluated.

### 3.4. Antiproliferation Activity

**Figure 6:**
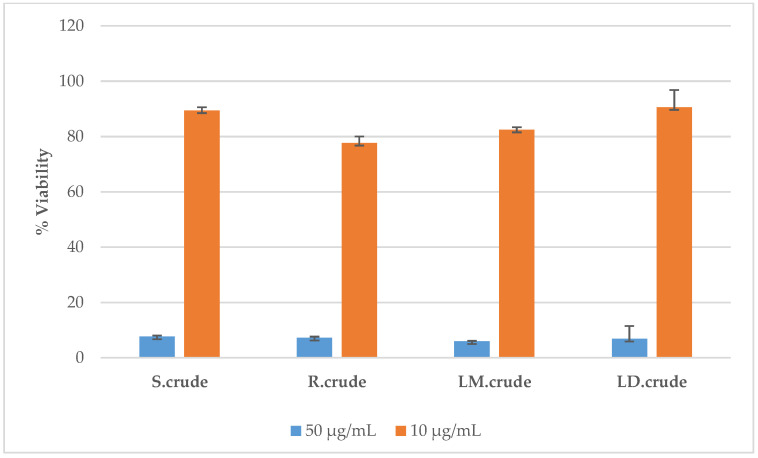
Antiproliferation activity against HeLa cells: S.crude—stem bark crude extract; R.crude—root crude extract; LM.crude—methanol leaf crude extract; LD.crude—dichloromethane leaf crude extract. Expressed as % parasite viability ± standard deviation.

**Figure 7:**
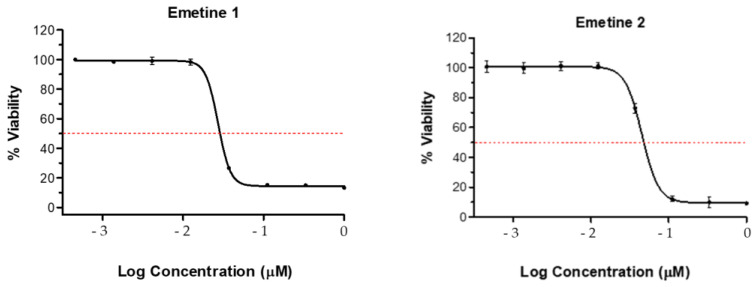
Antiproliferation activity against HeLa cells: the standard drugs emetine 1 and 2 at concentrations of 50 μg/mL and 10 μg/mL, expressed as percentage cell viability, respectively.

The cell toxicity assay (CTA) was used to determine the cytotoxic abilities of the crude extracts against HeLa (human cervix adenocarcinoma) cells. Briefly, HeLa cells were cultured with the respective samples for 48 h and the remaining percentage cell viability relative to untreated control cells determined using resazurin. All tested samples at a concentration of 50 µg/mL produced a significant cytotoxic effect against HeLa cells to below 50%, as shown in Figure 6. The cytotoxicity assay was repeated but at a lower concentration of 10 µg/mL, and none of the crude extracts showed significant cytotoxic effects, as shown in Figure 6. The standard drugs emetine 1 and 2 displayed cell viability of 0.027 μM and 0.045 μM, at concentrations of 50 μg/mL and 10 μg/mL, respectively (as shown in Figure 7). In the literature, a study has shown that *Breonadia salicina* has no toxic effects. The MeOH/CH_2_Cl_2_ (1:1) stem bark and leaf extracts did not affect the human kidney epithelial cells (70.84 ± 2.72 μg/mL and 182.66 ± 12.44 μg/mL, respectively) when using the 3-[4,5-dimethylthiazol-2yl]-2,5-diphenyltetrazolium bromide (MTT) assay [41]. This previous research report is in agreement with the results obtained in this study. In the literature, the antiproliferation activity of *Breonadia salicina* has not been determined using HeLa cells.

### 3.5. Genotoxicity

**Figure 8:**
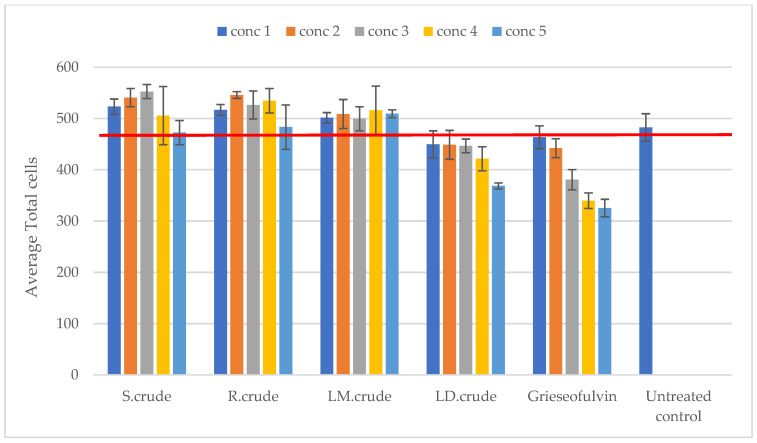
Genotoxicity against Vero cells: S.crude—stem bark crude extract; R.crude—root crude extract; LM.crude—methanol leaf crude extract; and LD.crude—dichloromethane leaf crude extract. Data (n = 4) expressed as total Vero cell number ± standard deviation at varying concentrations. The red line indicates the number of total cells in an untreated Vero population, i.e., a nontoxic treatment control. Concentrations: 1 = 15.125, 2 = 31.25, 3 = 62.5, 4 = 125 and 5 = 250 μg/mL.

**Figure 9:**
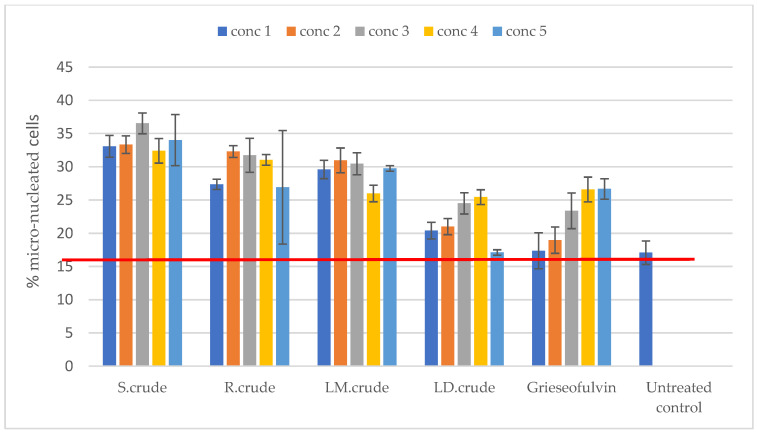
Genotoxicity against Vero cells: S.crude—stem bark crude extract; R.crude—root crude extract; LM.crude—methanol leaf crude extract; and LD.crude—dichloromethane leaf crude extract. Data (n = 4) expressed as percentage micro-nucleated Vero cells ± standard deviation at varying concentrations. The red line indicates the % micro-nucleated cells of the untreated Vero control population. Concentrations: 1 = 15.125, 2 = 31.25, 3 = 62.5, 4 = 125 and 5 = 250 μg/mL.

Genotoxicity was determined using the NucRed nuclei dye for the identification of micronuclei. The crude methanol leaf extract was not cytotoxic at concentrations of 15.125 μg/mL, 31.25 μg/mL, 125 μg/mL and 250 μg/mL. However, the crude dichloromethane leaf extract was cytotoxic at the highest tested concentration of 250 µg/mL, but did not cause cytotoxicity at the concentrations of 15.125 μg/mL, 31.25 μg/mL and 125 μg/mL, as shown in Figure 8. An increase in the percentage of micro-nucleated cells was evident with all crude extracts, indicating their potential genotoxicity, as shown in Figure 9. There was no increase observed with the crude dichloromethane leaf extract at 250 µg/mL, as shown in Figure 9. In the literature, the genotoxicity of *Breonadia salicina* has not been determined using Vero cells. Therefore, our study is the first to assess the genotoxicity of *B. salicina* against these cells.

### 3.6. Cytotoxicity

**Figure 10:**
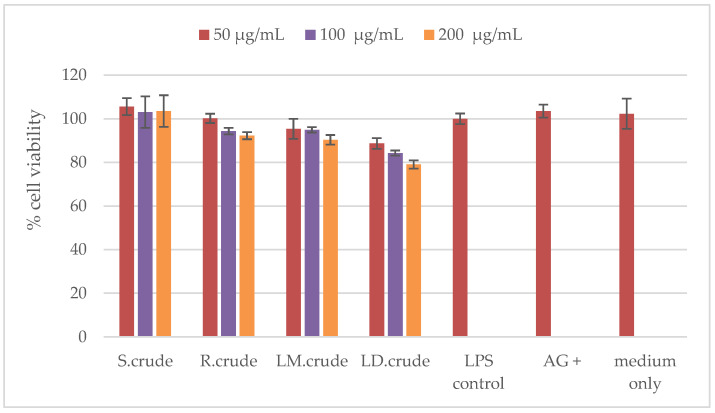
Cytotoxicity against RAW 264.7 macrophages: S.crude—stem bark crude extract; R.crude—root crude extract; LM.crude—methanol leaf crude extract; and LD.crude—dichloromethane leaf crude extract. Data (n = 4) expressed as percentage cell viability of LPS-activated macrophages ± standard deviation at varying concentrations of 50 μg/mL, 100 μg/mL and 200 μg/mL.

**Figure 11:**
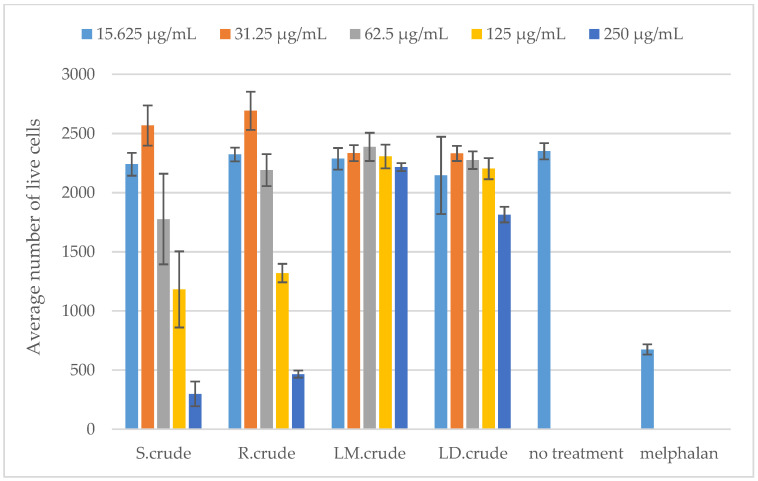
Cytotoxicity against Vero cells: S.crude—stem bark crude extract; R.crude—root crude extract; LM.crude—methanol leaf crude extract; and LD.crude—dichloromethane leaf crude extract. Data (n = 4) expressed as average number of live cells ± standard deviation at varying concentrations of 15.625 μg/mL, 31.25 μg/mL, 62.5 μg/mL, 125 μg/mL and 250 μg/mL.

The cytotoxic effect of the stem bark, root, methanol leaf and dichloromethane leaf crude extracts on RAW 264.7 macrophages was determined in order to accurately establish their potential anti-inflammatory activity using the MTT 3-(4,5-dimethylthiazol-2-yl)-2,5-diphenyltetrazolium bromide) assay. None of these extracts were cytotoxic at the concentrations of 50 μg/mL, 100 μg/mL and 200 μg/mL against RAW 264.7 macrophages (as shown in Figure 10). Furthermore, the cytotoxicity of the extracts was assessed using the Hoechst 33342/propidium iodide (PI) dual staining method against Vero cells (as presented in Figure 11). The crude stem bark and root extracts showed cytotoxic effects at a concentration of 250 μg/mL (as shown in Figure 11). The cytotoxic effect could be due to the synergistic effects of several compounds found in the crude stem bark and root extracts (as shown in Figure 2). Furthermore, Mahlo et al. (2013) reported that the chloroform leaf extract was less toxic to Vero monkey kidney cells (LC_50_ = 82 μg/mL) than ursolic acid (LC_50_ = 25 µg/mL) using the MTT (3-(4,5-dimethylthiazol)-2,5-diphenyl tetrazolium bromide) assay [3]. The stem bark and root extracts did not have cytotoxic effects at concentrations of 15.625 μg/mL, 31.25 μg/mL, 62.5 μg/mL and 125 μg/mL (as shown in Figure 11). Moreover, the crude leaf (methanol and dichloromethane) extracts were not cytotoxic against Vero cells at concentrations of 15.625 μg/mL, 31.25 μg/mL, 62.5 μg/mL, 125 μg/mL and 250 μg/mL (as shown in Figure 11). According to the literature, the toxicity of *Breonadia salicina* has not been determined using RAW 264.7 macrophages.

## 4. Conclusions

The main objective of this study was to determine the antidiabetic and anti-inflammatory activity, as well as the possible genotoxicity and cytotoxicity, of *Breonadia salicina.* This study provides a comprehensive analysis of the metabolites in different plant parts of *Breonadia salicina*. We found a wide range of different metabolites in the stem bark, root and leaf extracts of *Breonadia* species. Furthermore, this study confirms that molecular networking (MN) is a useful tool in the investigation of untargeted metabolomic spectral data. Future perspectives will involve the further mining of these metabolomes using more computational tools to discover new metabolites that could be potential natural products, and to describe methods that will assist in the more automated investigation of complex metabolomes. Furthermore, the study showed that the crude stem bark and root extracts exhibited more potent antidiabetic activity than the leaf extracts. The study further showed that the crude dichloromethane leaf extract had the highest anti-inflammatory activity compared to the stem and root extracts. In addition, the findings in this work comprehensively indicate that lipids, benzenoids, organic oxygen compounds, phenylpropanoids and polyketides contribute to the antidiabetic activity. The results of this study indicate that certain parts of *B. salicina* show cytotoxic effects against various panels of cells using different methods. However, it should be taken into consideration that in vitro results are not necessarily indicative of the possible outcomes in vivo. Therefore, all of the tested samples should be placed into an in vivo model to evaluate the toxicity of the metabolites. This study is the first to determine the phytochemicals contributing to the antidiabetic and anti-inflammatory activity of *Breonadia salicina* using a molecular networking approach.

## Data Availability

The data are contained within the article and Supplementary Materials. The data will be made available upon request.

## Supplementary Materials

The following supporting information can be downloaded at: https://www.mdpi.com/article/10.3390/metabo14060291/s1, Figure S1: UPLC-QTOF-MS chromatograms (negative mode electrospray ionization) of (A) S.crude—crude stem bark extract; (B) R.crude—crude root extract; (C) LM.crude—methanol leaf extract; and (D) LD.crude—dichloromethane leaf extract, Figure S2: UPLC-QTOF-MS chromatograms (positive mode electrospray ionization) of (A) S.crude—crude stem bark extract; (B) R.crude—crude root extract; (C) LM.crude—methanol leaf extract; and (D) LD.crude—dichloromethane leaf extract.
