# Supplementary material for: Molecular Networking-Based Metabolome, In Vitro Antidiabetic and Anti-Inflammatory Effects of Breonadia salicina (Vahl) Hepper & J.R.I. Wood
Source: Metabolites. 2024 May 21;14(6):291. doi: 10.3390/metabo14060291 (PMC11206052; doi:10.3390/metabo14060291)
Supplement: Supplementary file 1 [file metabolites-14-00291-s001.zip › metabolites-3017988-supplementary.pdf]

metabolites

MDPI

Supplementary material

Molecular Networking-based Metabolome, *In Vitro* Antidiabetic and Anti-inflammatory Effects of *Breonadia salicina* (Vahl) Hepper and J.R.I. Wood

Dorcas Tlhapi [1,*], Isaiah Ramaite [1], Chinedu Anokwuru [2] and Teunis van Ree [1]

[1] Department of Chemistry, Faculty of Science, Engineering and Agriculture, University of Venda, Private Bag X5050, Thohoyandou 0950, South Africa; Isaiah.Ramaite@univen.ac.za (I.D); Teuns.VanRee@univen.ac.za (T.v.R)
[2] Department of Basic Sciences, School of Science and Technology, Babcock University, Ilishan Remo 121103, Nigeria; anokwuruc@babcock.edu.ng (C.P)
* Correspondence: 11617811@mvula.univen.ac.za (D. Tlhapi)

Network analysis, *in silico* annotation and substructure annotation job links

1. Molecular network ESI(-):ID=c4e42d35fcf04dba9ce6fbb77074ec8e

https://gnps.ucsd.edu/ProteoSAFe/status.jsp?task=c4e42d35fcf04dba9ce6fbb77074ec8e

2. Network annotation propagation ESI(-):ID=41dd5777207242169a8ad9a53f99100f

https://gnps.ucsd.edu/ProteoSAFe/status.jsp?task=41dd5777207242169a8ad9a53f99100f

3. DEREPLICATOR ESI(-):ID=7d5ee752343f4dbc97890db08d0af00c

https://gnps.ucsd.edu/ProteoSAFe/status.jsp?task=7d5ee752343f4dbc97890db08d0af00c

4. MS2LDA ESI(-):ID=b12f963f218640e6ac648cbd513ac4a5

https://gnps.ucsd.edu/ProteoSAFe/status.jsp?task=b12f963f218640e6ac648cbd513ac4a5

5. MolNetEnhancer ESI(-):ID=d8cd4426b945492e96a1fc29e48070bc

https://gnps.ucsd.edu/ProteoSAFe/status.jsp?task=d8cd4426b945492e96a1fc29e48070bc

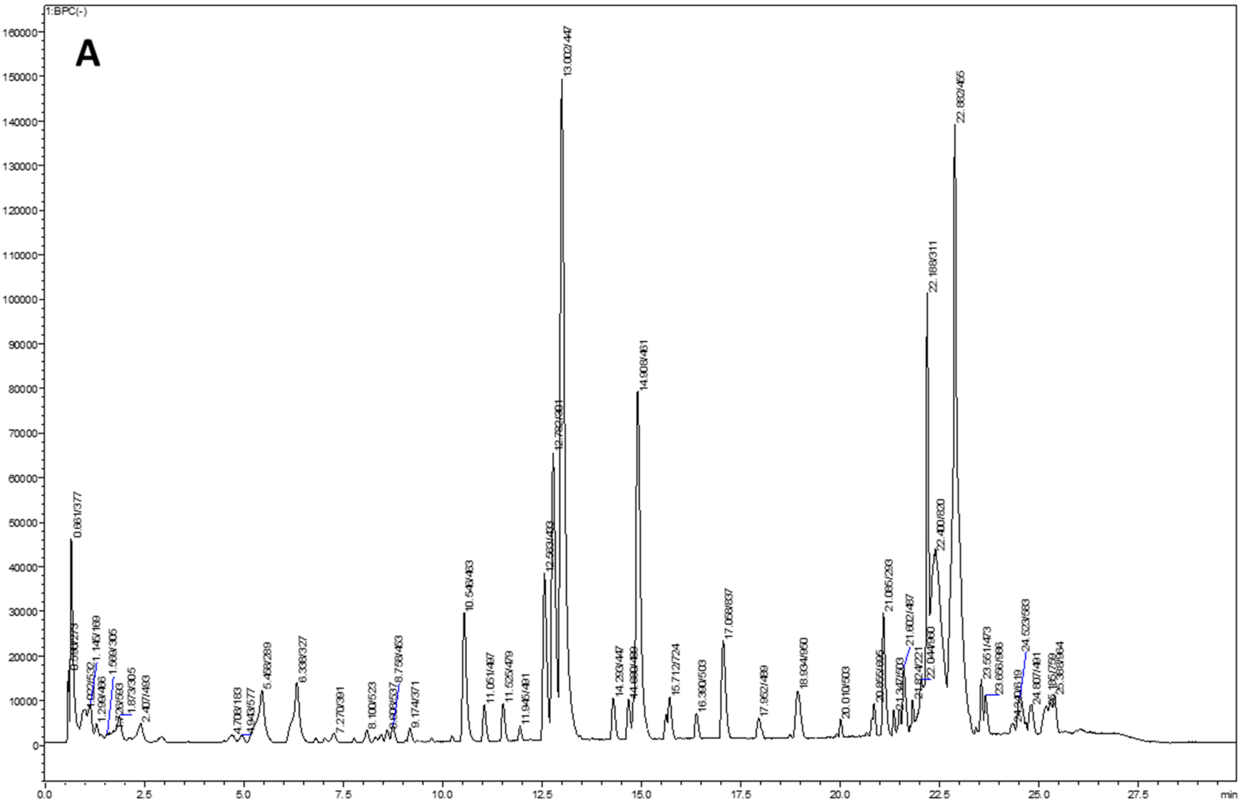

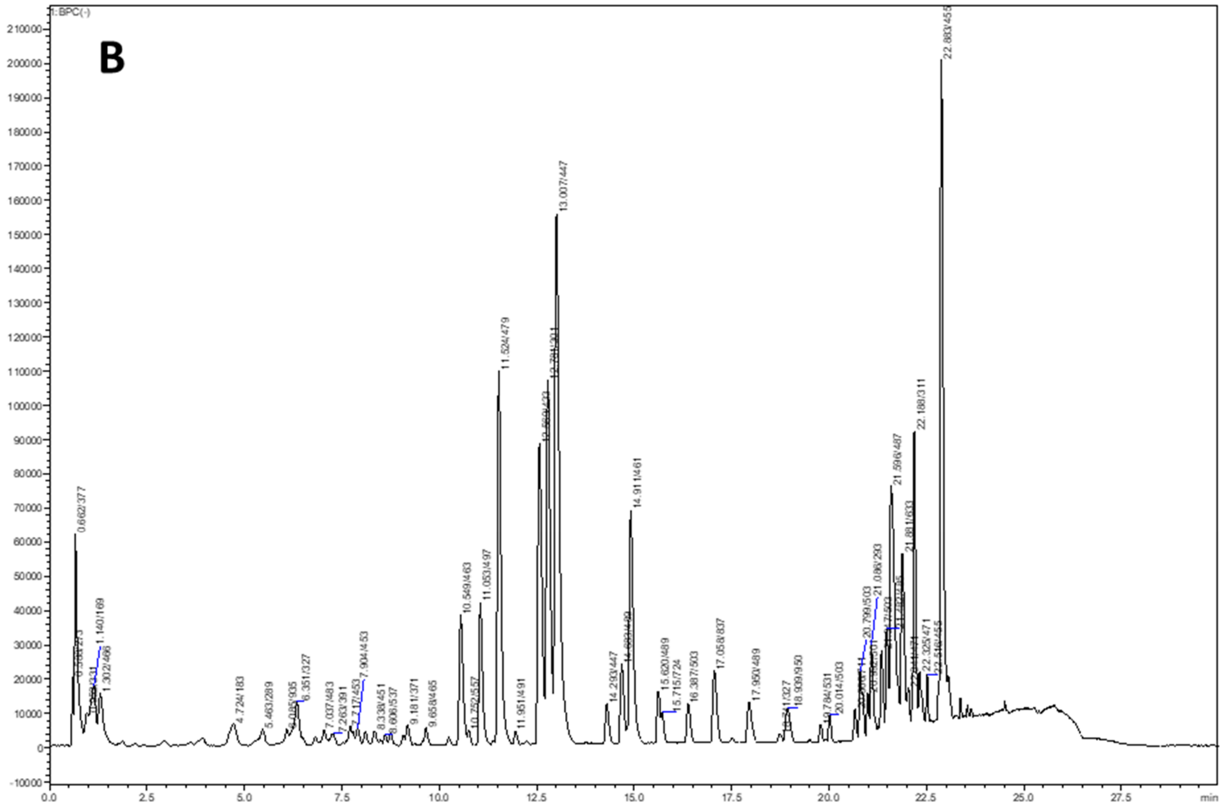
1:BPC(-)
B
min

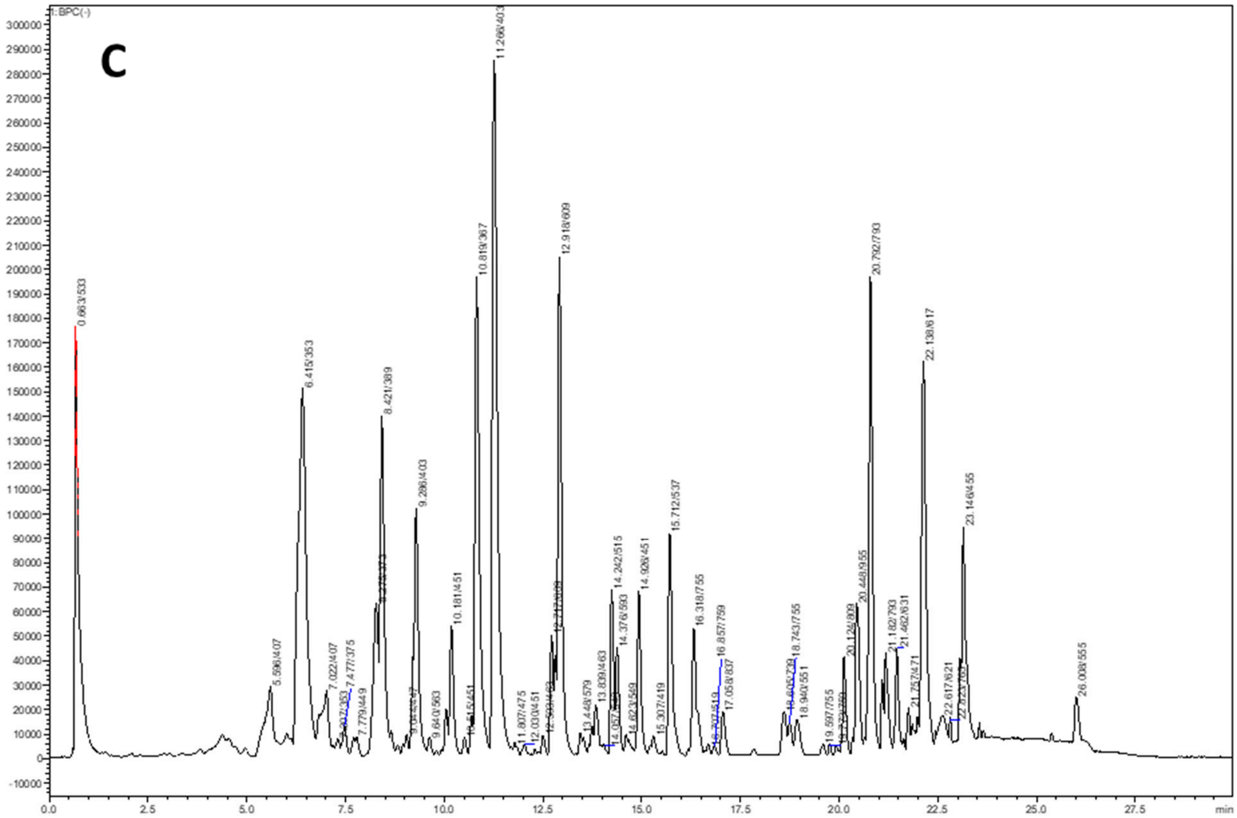
1:BPC(-)
C
min

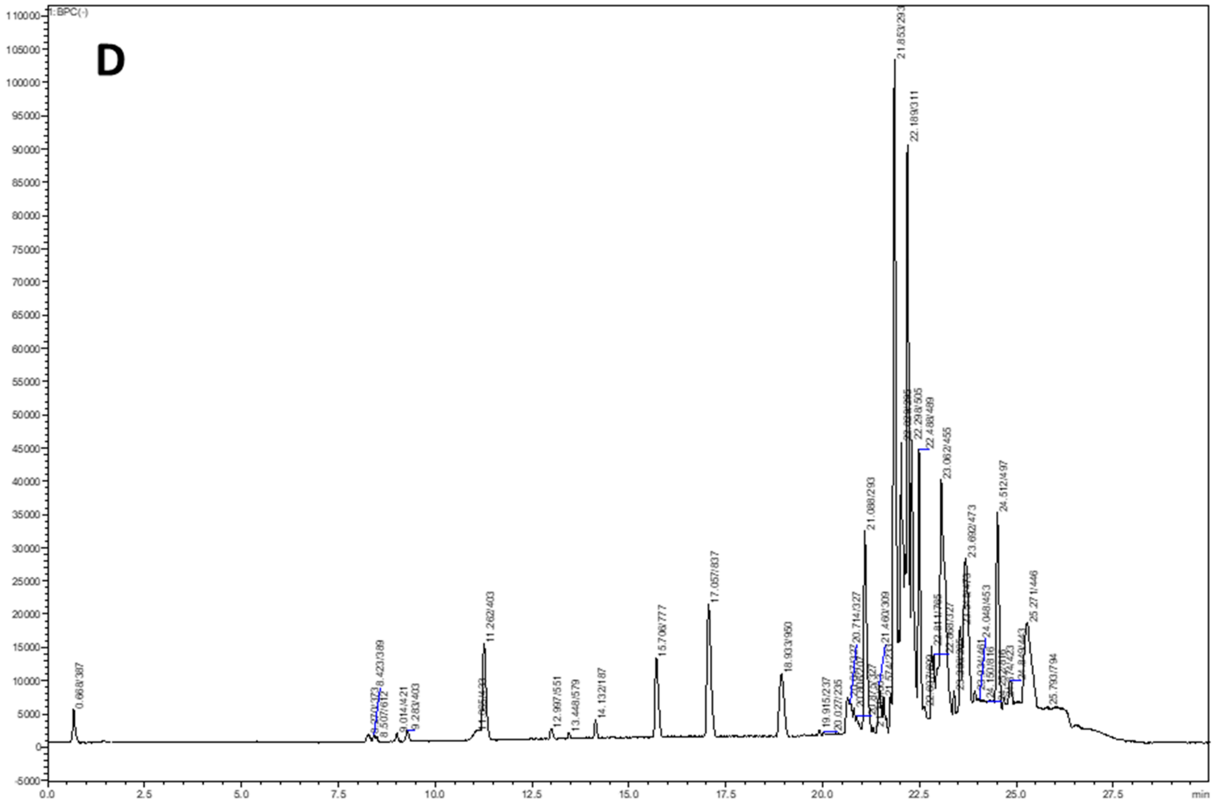

Figure S1. UPLC-QTOF-MS chromatograms (negative mode electrospray ionization) of (A) S.crude—crude stem bark extract; (B) R.crude—crude root extract; (C) LM.crude—methanol leaf extract; and (D) LD.crude—dichloromethane leaf extract.

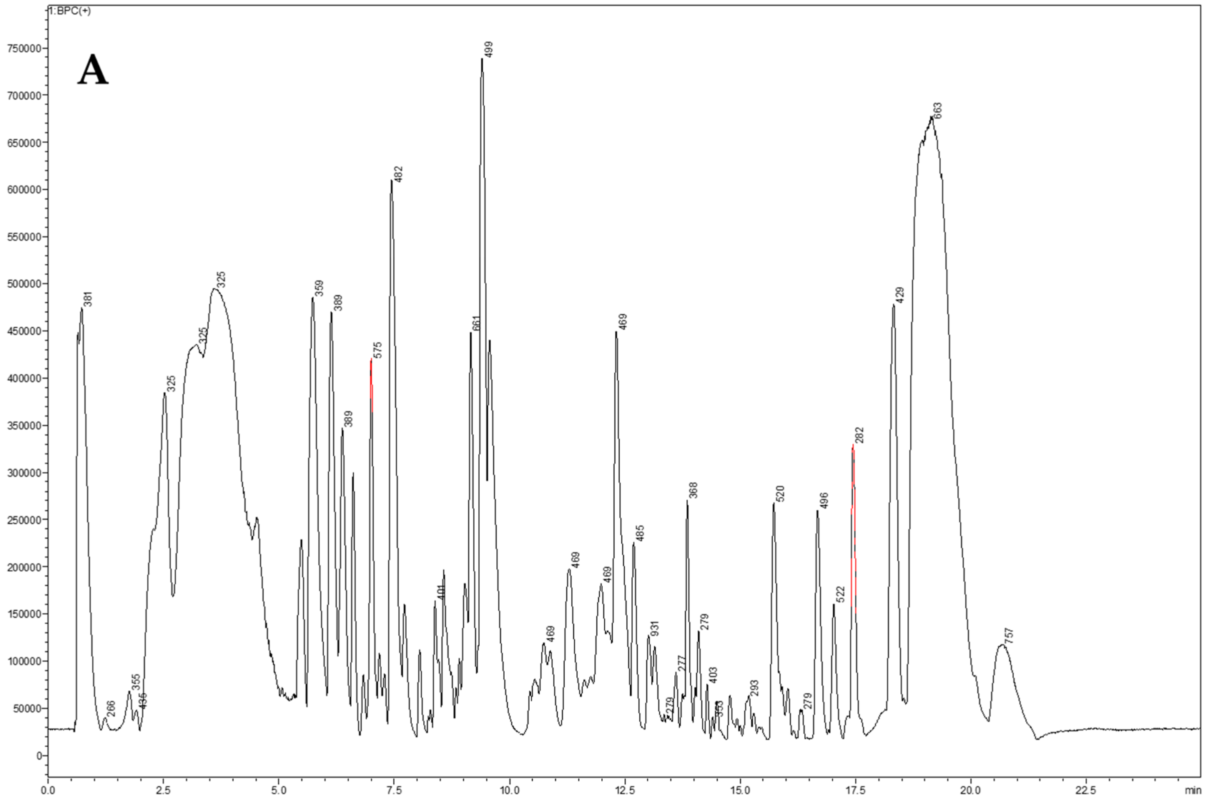
A
1:BPC(+)
750000
700000
650000
600000
550000
500000
450000
400000
350000
300000
250000
200000
150000
100000
50000
0
0.0
2.5
5.0
7.5
10.0
12.5
15.0
17.5
20.0
22.5
min

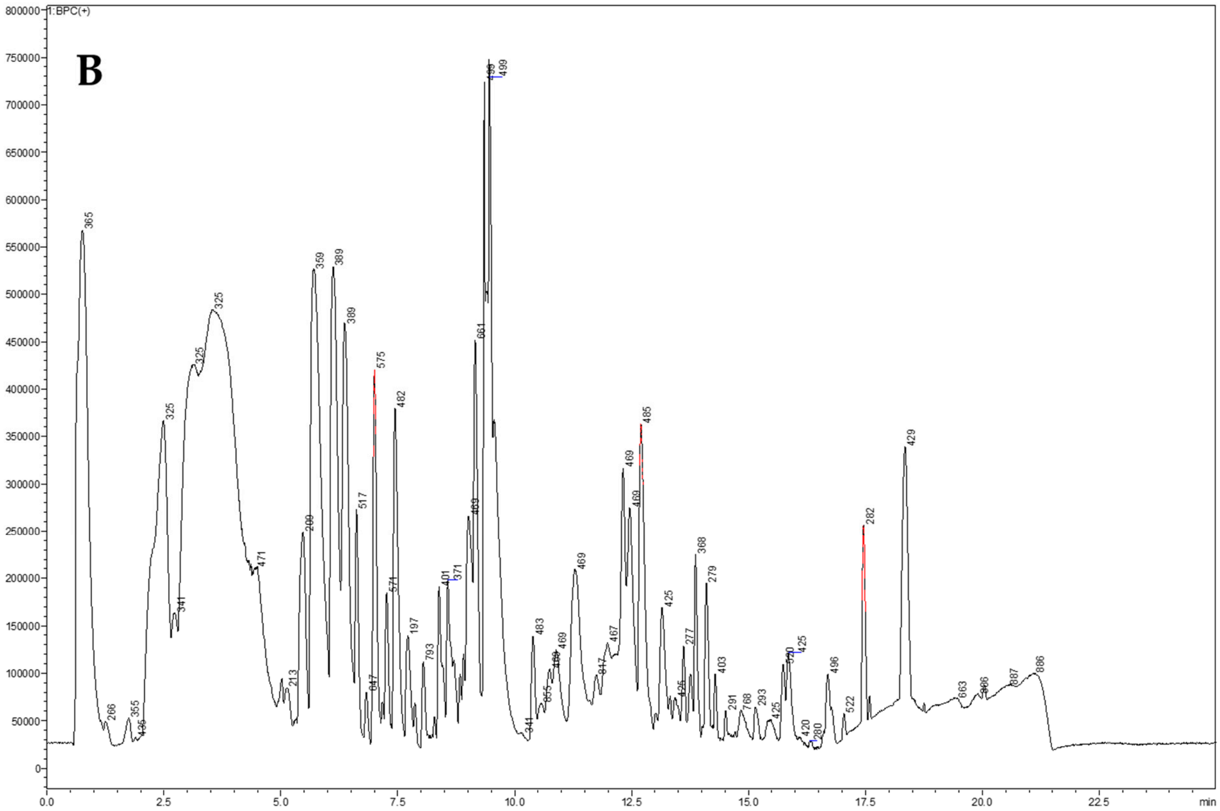
B
1:BPC(+)
800000
750000
700000
650000
600000
550000
500000
450000
400000
350000
300000
250000
200000
150000
100000
50000
0
0.0
2.5
5.0
7.5
10.0
12.5
15.0
17.5
20.0
22.5
min

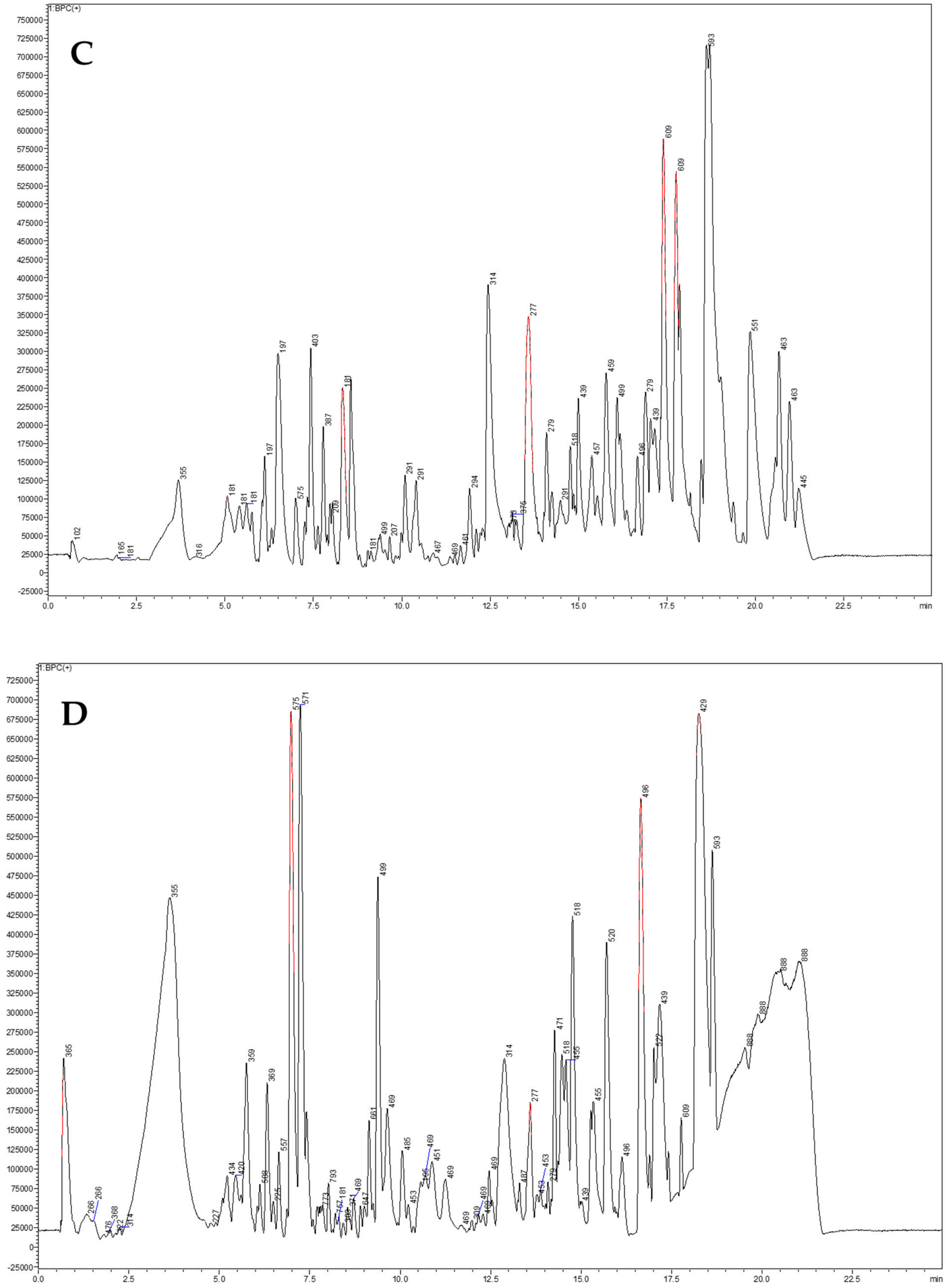

Figure S2. UPLC-QTOF-MS chromatograms (positive mode electrospray ionization) of (A) S.crude—crude stem bark extract; (B) R.crude—crude root extract; (C) LM.crude—methanol leaf extract; and (D) LD.crude—dichloromethane leaf extrac.
